# Phase-Shifted Fiber Bragg Grating by Selective Pitch Slicing

**DOI:** 10.3390/s24216898

**Published:** 2024-10-28

**Authors:** Paulo Robalinho, Vinícius Piaia, Liliana Soares, Susana Novais, António Lobo Ribeiro, Susana Silva, Orlando Frazão

**Affiliations:** 1 INESC TEC—Institute for Systems and Computer Engineering, Technology and Science, 4169-007 Porto, Portugal; up201800439@edu.fc.up.pt (V.P.); liliana.p.soares@inesctec.pt (L.S.); susana.novais@inesctec.pt (S.N.); susana.o.silva@inesctec.pt (S.S.); orlando.frazao@inesctec.pt (O.F.); 2 FEUP—Faculty of Engineering of the University of Porto, R. Dr. Roberto Frias, 4200-465 Porto, Portugal; 3 Faculty of Health Sciences, University Fernando Pessoa, 4200-150 Porto, Portugal; alobo@ufp.edu.pt

**Keywords:** optical fiber sensor, phase-shift FBG, fabrication

## Abstract

This paper presents a new type of phase-shifted Fiber Bragg Grating (FBG): the sliced-FBG (SFBG). The fabrication process involves cutting a standard FBG inside its grating region. As a result, the last grating pitch is shorter than the others. The optical output signal consists of the overlap between the FBG reflection and the reflection at the fiber-cleaved tip. This new fiber optic device has been studied as a vibration sensor, allowing for the characterization of this sensor in the frequency range of 150 Hz to 70 kHz. How the phase shift in the FBG can be controlled by changing the length of the last pitch is also shown. This device can be used as a filter and a sensing element. As a sensing element, we will demonstrate its application as a vibration sensor that can be utilized in various applications, particularly in monitoring mechanical structures.

## 1. Introduction

After the fiber optic gyroscope, one of the most commercially used fiber optic device for sensing has been the Fiber Bragg Grating (FBG). This optical device was first demonstrated by Ken Hill in 1978 and was fabricated using visible light injected into the Ge-doped core of an optical fiber, which induced refractive-index changes in its core [1]. In 1989, Gerald Meltz presented a new fabrication method that was based in transverse holographic inscription by external side writing the grating using a bulk glass phase mask and ultraviolet light illumination, ushering the era of FBGs as sensing elements and as wavelength-specific dielectric mirrors for fiber lasers [2]. More recently, the introduction of femtosecond lasers has made possible the fabrication of more robust FBGs, allowing them to be used in harsh conditions [3].

The FBG manufacturing process modulates the refractive index profile of the optical fiber [4]. This modulation is achieved by exposing the fiber to a pulsed UV laser [5] using three techniques: interferometry [6], phase masking [7], and point-to-point inscription (direct writing) [8]. Through interferometry, the induction of refractive index modulation in the fiber core results from the spatial interferometric pattern generated by the superposition of a UV light beam [9]. Although currently uncommon to implement, this method has the advantage of requiring a single pulse on short gratings and the disadvantage of calibration complexity. The use of phase masks is the most common method [10,11]. This method consists of the transverse exposure of the optical fiber to the periodic spatial pattern generated by the diffraction of the UV light beam by the phase mask. This technique allows for greater fractionation stability; however, different FBGs require different phase masks. The third method is direct writing [12,13,14]. In this case, each perturbation of the refractive index profile is written individually. Typically, the speed and displacement of the beam along the fiber is dependent on the laser repetition rate. The advantage of this method is the wider range of refractive index profiles induced in the optical fiber, but it requires a longer duration than the two techniques mentioned above.

FBGs can be classified into different structure configurations [15]. See Table 1. The uniform configuration consists of a constant period of positive-only refractive index modulation. This can optically reflect a narrow band of light that varies with temperature and strain due to changes in the optical paths and effective refractive index modulation. It typically has temperature and strain sensitivities of 10 pm/°C and 1 pm/με, respectively. Apodized grating, although the modulation period of the refractive index remains constant, the refractive index profile follows a Gaussian or high-cosine geometry, resulting in a lower intensity of index modulation at the ends compared to the central region [16,17,18]. This feature allows the side lobes in the reflection spectrum to be attenuated, improving the spectral signal-to-noise ratio. Chirped-FBG (CFBG) consists of a non-uniform refractive index modulation, with the period normally increasing during the manufacturing process [19]. This change allows the reflected light band to be broadened [20,21]. The Tilted-FBG (TFBG) consists of a standard FBG with perturbations at an angle to the propagation axis [22]. This modification excites cladding modes, making the grating sensitive to the external refractive index [23,24,25]. Etched FBG (EFBG) is another way to increase the sensitivity of the FBG to environmental conditions [26]. In this case, part of the fiber cladding is chemically removed to reduce the diameter of the optical fiber, thus decreasing the confinement. FBGs can be fabricated in groups along the fiber, allowing multiple parameters to be studied simultaneously; this configuration is known as an FBG array. Additionally, by varying the phase profile along the FBG, it is possible to obtain small rejection bands within the reflected light band [27]. One of the sensing applications of phase-shift FBGs (PSFBG) is mechanical vibration monitoring. FBGs can be used to determine the vibrations of a given mechanical structure up to the kHz range, and using phase-shifted FBGs improves the signal-to-noise ratio (SNR) compared to standard FBGs [28]. In addition, the application of FBG at the tip of the optical fiber as an anti-reflective system has been theoretically modeled [29].

There are several ways to interrogate a phase-shift FBG, from the use of broad-spectrum sources to lasers, as well as, from the use of optical spectrum analyzers (OSA) or spectrometers to photodetectors [30,31]. An OSA or spectrometer allows the phase of the system to be determined, but these are slow measurements [32]. Using photodetectors, on the other hand, allows for faster measurements where the FBG sensor models the signal intensity. In the latter case, a wide-spectrum source can be used, which implies the need to implement other FBGs to function as optical filters [33,34]. In addition, the implementation of a photodetector can also be combined with a tunable laser where only one sensor FBG is required [35], which is the configuration used in this work for the vibration measurement, in a reflection configuration. Some applications of FBGs as vibration sensors are shown in Table 2.

The aim of this paper is to present the new phase-shift FBGs obtained by cleaving the FBG within its physical length (Table 1). The reflected output signal is the overlap of the FBG reflection spectra combined with its transmission spectra, which is reflected at the tip of the cleaved fiber and again transmitted through the FBG. Due to the cut within the index, modulation pitch of the FBG, it is possible to control the phase shift as shown below. The new device is demonstrated as a vibration sensor.

## 2. Numerical Model

A standard FBG is characterized by uniform modulation in the fiber’s core refractive index, creating a sequence of interface reflection points. When an incident electromagnetic plane wave interacts with this optical structure, the resulting reflective electromagnetic wave can be described by Equation (1). This arises from the constructive interference of the various waves that are reflected inside the device.

(1) $$E_{r}=Er_{\Lambda }\sum_{k=0}^{N}{\left(t_{\Lambda }e^{i\beta L_{\Lambda }}\right)}^{2k}=\frac{Er_{\Lambda }(1-{\left(t_{\Lambda }e^{i\beta L_{\Lambda }}\right)}^{2N+2})}{\left(1-{\left(t_{\Lambda }e^{i\beta L_{\Lambda }}\right)}^{2}\right)}$$

where β is the propagation coefficient within the fiber and is given by *β* = 2*πn_eff_*/*λ*, *n_eff_* is the effective core refractive index, *λ* is the wavelength of the light being propagated in the fiber core, N is the number of interface reflective points, $L_{\Lambda }$ is the length between these reflective points and *t*_Λ_ and *r*_Λ_ are the transmission and reflection coefficient of which reflection points, respectively, *t*_Λ_ = (*2n*_1_*n*_2_)/(*n*_1_ + *n*_2_) and *r*_Λ_ = (*n*_1_ − *n*_2_)/(*n*_1_ + *n*_2_). In addition, the transmitted wave can be mathematically described by Equation (2).

(2) $$E_{t}=E{t_{\Lambda }}^{N+1}e^{i\beta NL_{\Lambda }}$$

**Figure 1:**
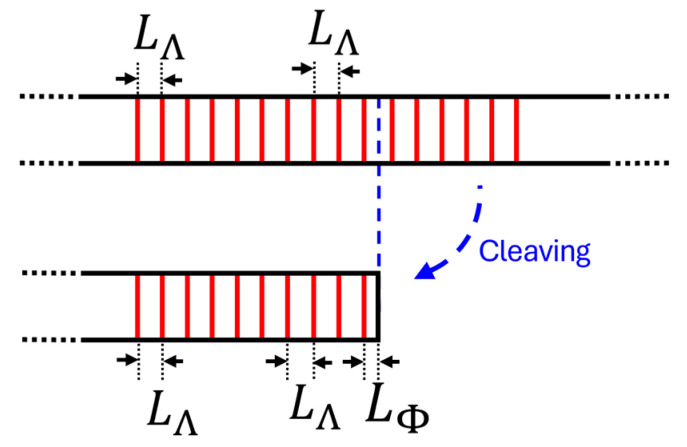
SFBG manufacturing scheme.

The device under study consists of a standard FBG cleaved into two parts (Figure 1).

Cleaving the standard FBG into two parts implies that the last pitch of the structure (delimited by the last interface perturbation of the FBG and the end of the cleaved optical fiber) has a shorter length than the other pitches. This feature allows the introduction of a phase shift in the output spectrum dependent on the size of the last pitch. The size of the structure is constrained by its fabrication to a value between 0 and *L*_Λ_. Thus, the output wave can be described mathematically by the superposition of the reflected wave by the FBG and the reflected wave at the tip of the fiber and transmitted twice by the FBG (Equation (3)).

(3) $$E_{r}=E\left[\frac{r_{\Lambda }(1-{\left(t_{\Lambda }e^{i\beta L_{\Lambda }}\right)}^{2N+2})}{\left(1-{\left(t_{\Lambda }e^{i\beta L_{\Lambda }}\right)}^{2}\right)}+R_{\Phi }{t_{\Lambda }}^{2N+2}e^{i\beta (2L_{\Phi }+2NL_{\Lambda })}\right]$$

**Figure 2:**
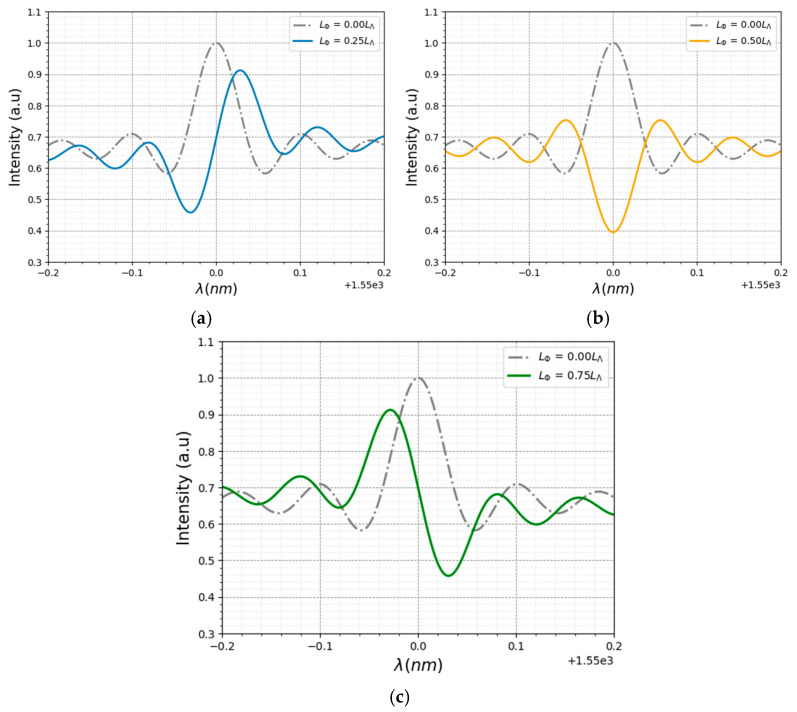
The graphical representation of Equation (3) simulation for: (**a**) *L*_Φ_ = 0, *L*_Φ_ = *L*_Λ_/4, (**b**) *L*_Φ_ = *L*_Λ_/2, (**c**) *L*_Φ_ = 3*L*_Λ_/4.

**Figure 3:**
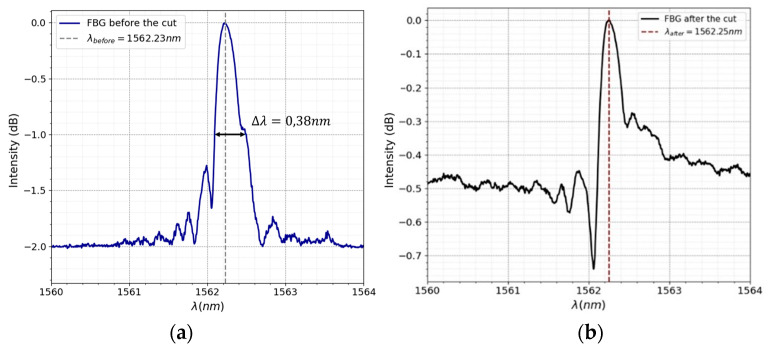
The spectrum of FBG structure: (**a**) before the cut and (**b**) after the cut.

The *L*_Φ_ is the key parameter for controlling the geometry of the spectrum. Figure 2 is a computer simulation of the spectrum obtained for four different values of *L*_Φ_, where the following parameters are used: *λ_B_* = 1550 nm, length of 10 mm and *n_eff_* = 1.46 and Δ*n* = 8.3 × 10^−4^ (Equation (4)) [41].

(4) $$FWHM=\lambda_{B}s\sqrt{{\left(\frac{\Delta n}{2n_{eff}}\right)}^{2}+{\left(\frac{1}{N}\right)}^{2}}$$

where the quantity *s* is indicative of the power of the grating, with a value of ~1 denoting a strong grating and a value of ~0.5 denoting a weak grating. The full width at half maximum (*FWHM*) was obtained with the spectrum of the FBG prior to cleaving (see Figure 3a).

For *L*_Φ_ = 0, the structure is the same as a standard FBG, since there is no different pitch length to the others, which implies similarity in the spectrum achieved (band reflect filter). When 0 < *L*_Φ_ < *L*_Λ_/2, the main minimum appears at a shorter wavelength than the maximum of the spectrum. Both the spectrum maximum and minimum move to higher wavelengths as the value of *L*_Φ_ increases. When *L*_Φ_ = *L*_Λ_/2, the spectrum maximum disappears and only the minimum remains. In this case, the spectrum has the shape of a band reject. For *L*_Λ_/2 < *L*_Φ_ < *L*_Λ_, the maximum reappears at a wavelength shorter than the central wavelength, and both the main maximum and minimum of the spectrum have a red shift. For *L*_Φ_ = *L*_Λ_, the same spectrum is obtained as in the case of *L*_Φ_ = 0. The above-mentioned pitch length sensibility also permits the establishment of a correlation between the peak differences and the tension applied to the SFBG, which may be considered as one of the possible applications. The simulations demonstrated that this phenomenon also occurs for multiples of *L*_Λ_. However, it was observed that as the space increases, the interaction between the two waves decreases, resulting in a reduction in the observed effect and a convergence toward the expected FBG reflected signal.

The SFBG can be used both as a sensor, for applications such as vibration and strain sensing, as well as an optical filter for use in optical interrogators. Additionally, a methodology for creating an anti-reflection FBG was identified in the literature. To achieve this, it was necessary to control the length of the pitches and the perturbation of the refractive index, with the objective of obtaining a size of a quarter of the wavelength. This approach would result in a reduction in Fresnel reflection through the application of destructive interference [29].

## 3. Experimental Results

An FBG was experimentally characterized using an optical broadband source with a spectral bandwidth of 100 nm (FWHM) centered at 1550 nm, a three-port optical circulator (Thorlabs 6015-3) to monitor the FBG reflected spectra, and an OSA instrument (model YOKOGAMA AQ6370C, Osaka, Japan). An 8 mm length FBG with a Bragg wavelength at 1562.23 nm and reflectivity of 2 dB manufactured by FiSens, was used. Its spectrum is shown in Figure 3a. The FBG was cut approximately in half, and Figure 3b shows the correspondent spectrum. The precision of the cut is achieved by using marks made during the manufacture of the FBG and by using a microscope to help position the cutting blade. The appearance of a minimum is observed, which, given that it occurs at a shorter wavelength than the wavelength at which the maximum is located, means that the last pitch length is less than half of the length of the other pitches.

**Figure 4:**
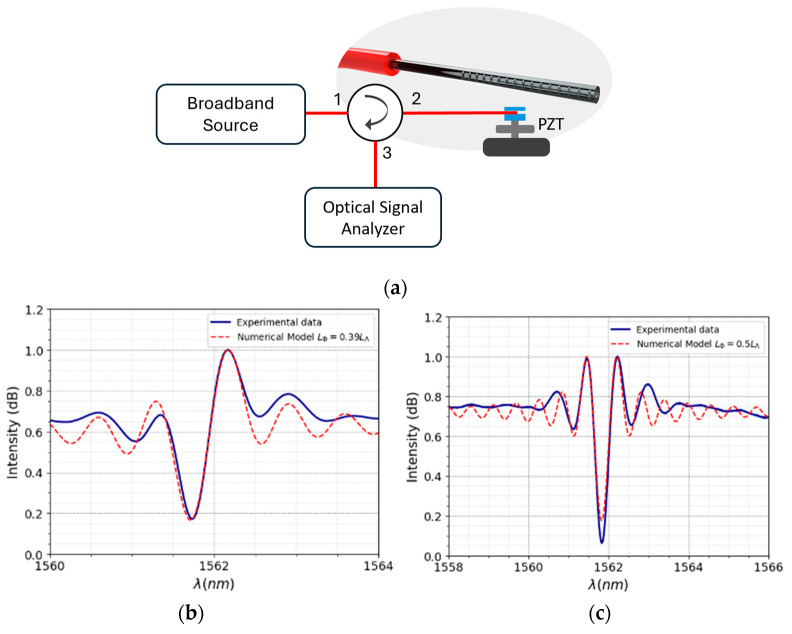

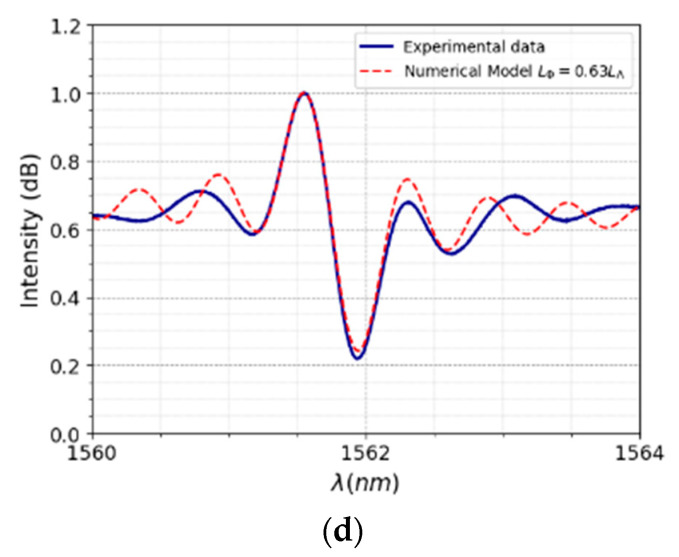
(**a**) Scheme used for the spectral analysis, (**b**) *L*_Φ_ < *L*_Λ_/2, (**c**) *L*_Φ_ = *L*_Λ_/2 and (**d**) *L*_Φ_ > *L*_Λ_/2. These results were obtained from the SFBG, and the variation is due to the applied axial stress.

The SFBG was then characterized as a vibration sensor. To optimize the mechanical vibration coupling of the oscillator to the optical fiber, it was necessary to radially pre-tension the fiber, and this results in a change in the ratio between the size of the last pitch and the remaining pitch, leading to a variation in the output spectrum (Figure 4a). Changing the pressure applied to the structure through the fixing screw allows an experimental demonstration of the hypotheses formulated in the theoretical model: *L*_Φ_ < *L*_Λ_/2 (on Figure 4b), *L*_Φ_ = L_Λ_/2 (on Figure 4c), and *L*_Φ_ > *L*_Λ_/2 (on Figure 4d).

**Figure 5:**
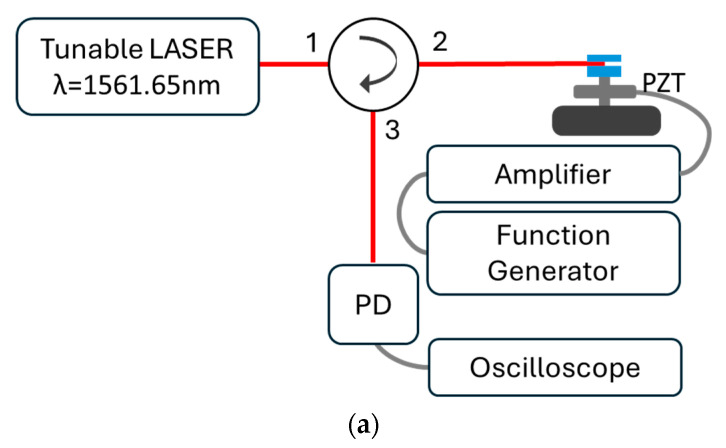

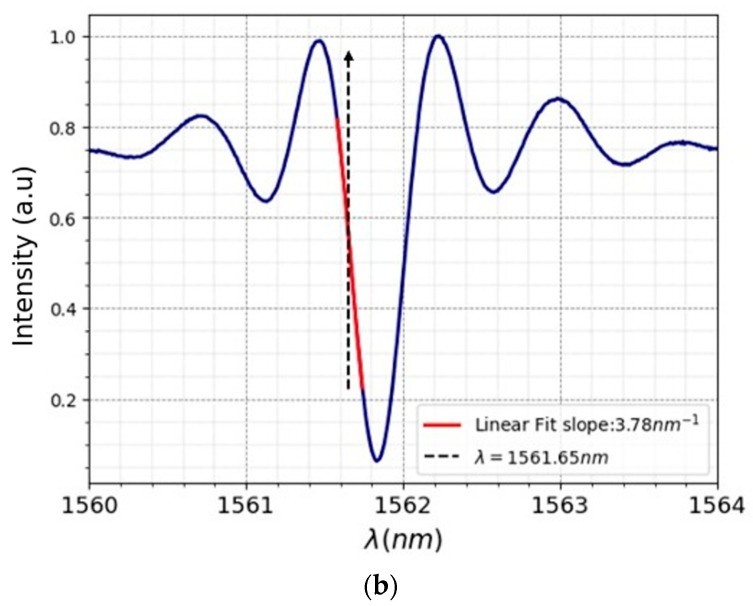
(**a**) Scheme for the FBG’s vibration characterization, (**b**) spectrum used for the measurement.

The system characterization for mechanical vibration is schematically shown in Figure 5a. Considering the need for sampling that allows the measurement of temporal signals with spectral components of the order of 100 kHz, a tunable laser (SANTEC), an optical circulator, a photodetector (PDA10CS-EC from Thorlabs), and a digital oscilloscope (GW-INSTEK, model GDS-2304A) were used. Furthermore, for a vibration generator, a piezoelectric transducer, abbreviated as PZT (Physik Instrument, model 80487) that was driven by a high-voltage amplifier (TEGAM, model 2350) and a signal function generator (SRS, model DS345), was used. The pressure applied to the SFBG was calibrated to achieve the condition *L*_Φ_ = *L*_Λ_/2. A wavelength of 1561.65 nm was used to interrogate the sensor. At a range of 0.17 nm, the spectrum shows a linear intensity variation of wavelength with a slope of −3.78 ± 0.02 nm^−1^ with r^2^ = 0.9996 (Figure 5b). Finally, a sinusoidal signal with an amplitude of 50 V and an offset of 40 V was applied to the PZT, and the frequency band from 150 Hz to 70 kHz was used.

**Figure 6:**
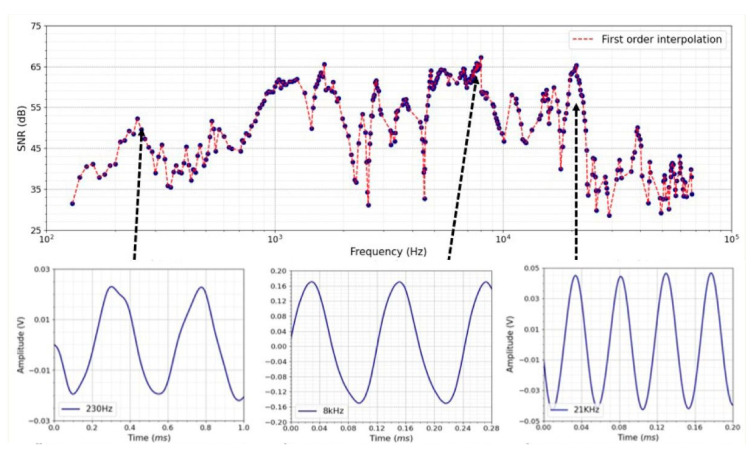
SNR versus frequency plots revealed a range of responses between 0.15 and 70 kHz. Below are the signals acquired by the photodetector when the oscillator oscillates at 230 Hz, 8200 Hz and 21 kHz and the corresponding Fourier transforms.

Figure 6 shows the characterization of the entire system as a function of the oscillator frequency. The different signals measured by the photodetector allow us to conclude the existence of resonance and anti-resonance resulting from the different connection pieces between the oscillator and the optical fiber. The system can measure frequencies from 0.15 kHz to 75 kHz with a minimum SNR of 30 dB, and it is most likely to oscillate in the frequency range from 1 kHz to 21.5 kHz, with the main resonance located at 8.20 ± 0.01 kHz associated with an SNR of 67 dB. There are also secondary resonances at 1.75 ± 0.01 kHz, with an SNR of 52 dB, and 21.0 ± 0.7 kHz with an SNR of 65 dB.

## 4. Conclusions

This paper presents a new approach to manufacturing a phase-shifted FBG. In this case, the phase shift is introduced by cutting a standard uniform FBG within a given pitch. By controlling the dimension of the last pitch, it is possible to vary the location of the phase shift and improve the intensity ratio between the minimum and maximum of the spectrum. Furthermore, since the phase shift is obtained by the last pitch, bounded by the last perturbation of the FBG and the end of the cleaved optical fiber, the intensity of the main minimum of the spectrum depends on the external refractive index (which is not the case with the traditional phase-shift FBG). As an application, the device has been tested as a vibration sensor and allows measurements within the frequency range between 150 Hz and 70 kHz. In addition to being smaller and easier to manufacture, its final spectral response can be tailored, making it an attractive device both as an optical filter and as a sensor. The main applications of the SFBG structure are in buildings and bridges, where it is possible not only to monitor the strains and vibrations to which they are subjected but also to determine water infiltration in the foundations of the structures. The sensor can also be used in the field of seismology or on farms to control access by people or vehicles and to monitor soil moisture, as well as to monitor chemical or biomedical parameters using thin films applied to the structure.

## Figures and Tables

**Table 1:** Different FBGs Structures.

Structure	Modulation Design
Uniform FBG Periodic modeling of the refractive index of the optical fiber core.	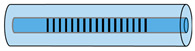
Apodized FBG FBG Uniform with a second modulation.	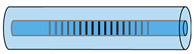
Chirped FBG The spacing between each disturbance is not uniform. It is typically increases in a given direction.	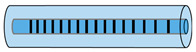
Tilted FBG Refractive index disturbances with a non-zero angle to the transverse axis of propagation.	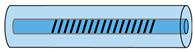
FBG Array Group of FBGs in the same optical fiber.	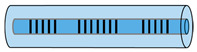
Phase-Shift FBG One or spacing between disturbances is similar (not equal) to the most recurrent value.	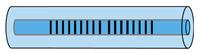
Slice FBG (In this work) The FBG is sliced/cleaved in two, resulting in the last pitch being shorter than the others.	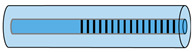

**Table 2:** Applications of FBGs as vibration sensors.

Reference	Dynamic Bandwidth (kHz)	SNR (dB)	Configuration
[36]	0–1	32	Transmission
[37]	0–0.1	29	Reflection
[38]	10–1000	30	Both direction simultaneous
[30]	0–16	30	Reflection
[39]	0–6	25	Reflection
[40]	0–1000	43	Both direction
This Work	0–75	Min 35|Max 65	Reflection

## Data Availability

The data are not publicly available due to confidentiality.
